# AnovArray: a set of SAS macros for the analysis of variance of gene expression data

**DOI:** 10.1186/1471-2105-6-150

**Published:** 2005-06-16

**Authors:** Christelle Hennequet-Antier, Hélène Chiapello, Karine Piot, Séverine Degrelle, Isabelle Hue, Jean-Paul Renard, François Rodolphe, Stéphane Robin

**Affiliations:** 1 Unité Mathématique, Informatique et Génome, INRA, 78352 Jouy-en-Josas, France; 2 INA-PG / INRA 16 rue Claude Bernard, F-75231 PARIS cedex 05 – France; 3 Unité Biologie du Développement et de la Reproduction, INRA, 78352 Jouy-en-Josas, France

## Abstract

**Background:**

Analysis of variance is a powerful approach to identify differentially expressed genes in a complex experimental design for microarray and macroarray data. The advantage of the anova model is the possibility to evaluate multiple sources of variation in an experiment.

**Results:**

AnovArray is a package implementing ANOVA for gene expression data using SAS^® ^statistical software. The originality of the package is 1) to quantify the different sources of variation on all genes together, 2) to provide a quality control of the model, 3) to propose two models for a gene's variance estimation and to perform a correction for multiple comparisons.

**Conclusion:**

AnovArray is freely available at  and requires only SAS^® ^statistical software.

## Background

Macroarray and microarray experiments are powerful technologies to simultaneously study thousands of genes. These technologies are efficient to identify differentially expressed genes between two or more samples (conditions). However the complexity of experiments may require an experimental design including different kinds of repeats. Analysis of variance (ANOVA) is a well suited statistical method to analyse gene expression depending on several sources of variation ([1-3]). It permits to decide which effects are important and to estimate them. One great interest of anova is to estimate the variations due to the gene factor taking into account interactions with other experimental factors.

Recently, several tools have been developped to perform anova on gene expression data (library YASMA [4] written in R, MAANOVA [5] written in R and Matlab, GeneANOVA [6] written in JAVA,...). Compared to these tools, we propose three improvements : a control quality procedure, a choice of two models for gene's variance estimation, and an additional correction for multiple comparisons.

## Implementation

### Language selection

The AnovArray package is a flexible tool to perform analysis of variance. It is developped in SAS^® ^statistical language in order to take advantage of statistical capabilities and powerful data management. Thanks to the SAS^® ^environment, AnovArray makes it possible to quantify gene effect and their interactions with other factors assuming all genes have the same variance. The anova method implemented in R is based on a matrix inversion and could not produce results on a large dataset with thousands of genes. For balanced factorial designs, the anova method implemented in SAS^® ^provides the anova table and some statistics (means, tests of effects,. . .). Our package uses these statistics and gives additional ones in order to quantify and estimate the factors, and also to identify differentially expressed genes. Moreover, AnovArray package benefits of all SAS^® ^possibilities (clustering, supervised classification, singular value decomposition, regression, etc...) for further analyses.

### Models of anova

AnovArray is a set of five SAS^® ^macros: **global_analysis, adjust, cleandata, differential_analysis and comparison**. It is dedicated to analysis of variance in the case of a balanced experimental design. A user-defined analysis of variance model is used for the macros global_analysis and differential_analysis. In the macro global_analysis, the anova model includes factor "Gene" and their interactions with other factors. It is performed on all genes together making the model more powerful to estimate experimental factors than if the model was defined for each gene. If necessary, several models can be considered by the user in order to test the impact of possible experimental factors.

To identify differentially expressed genes, AnovArray offers the choice between two variance models: an homogeneous model (HOM) and an heterogeneous model (HET). All genes have the same variance in the homogeneous model whereas each gene has its own variance in the heterogeneous model. If the hypothesis HOM is acceptable, the power of differentially expressed genes detection is greater, especially when only few repeats are available.

### Quality control

**Figure 2:**
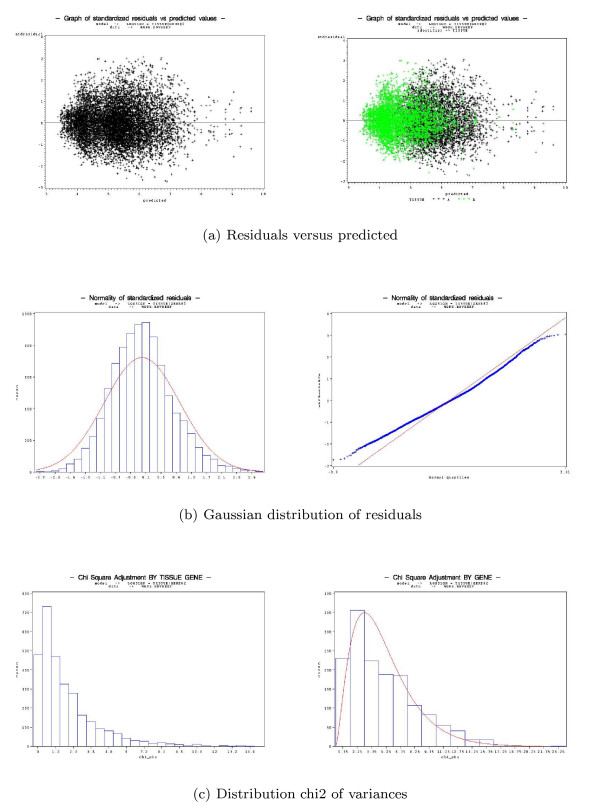
**Graphs produced by the macro global_analysis**. Residuals versus predicted, Gaussian distribution and Distribution chi2 of variances.

AnovArray contains facilities to evaluate the quality of the anova model defined by the user in the macro global_analysis. First, the classical anova table gives an estimation of the variability due to each factor. This table permits the user to classify the most important factors. Second, the model assumptions can be checked graphically (figure 2) by the user. The model assumes that residuals follow a gaussian distribution, are independent and have the same variance. Typically, figure 2a, permits to check that the residuals close to zero and do not show any structure. Figure 2b describes two kinds of graphs to check the residuals distribution: by fitting a gaussian distribution on residuals histogram and by using a Q-Q plot (plot of residuals distribution quantiles versus gaussian distribution quantiles). The hypothesis that residuals have the same variance (and also genes) is checked by fitting a chi-squared distribution (figure 2c). These graphs can also be very useful to describe which experimental factor affects particular genes. If the model is well adapted, the user could carry on the identification of differentially expressed genes. If not, the user has either to identify a subgroup of genes which do not satisfy the assumptions of the model (atypical genes), or to reconsider the factors included in the model. So thanks to AnovArray, it is possible to correct undesirable effets identified in the graphical representation using adjust macro and to remove a group of genes having an atypical behaviour using cleandata macro.

### Way of use

**Figure 1:**
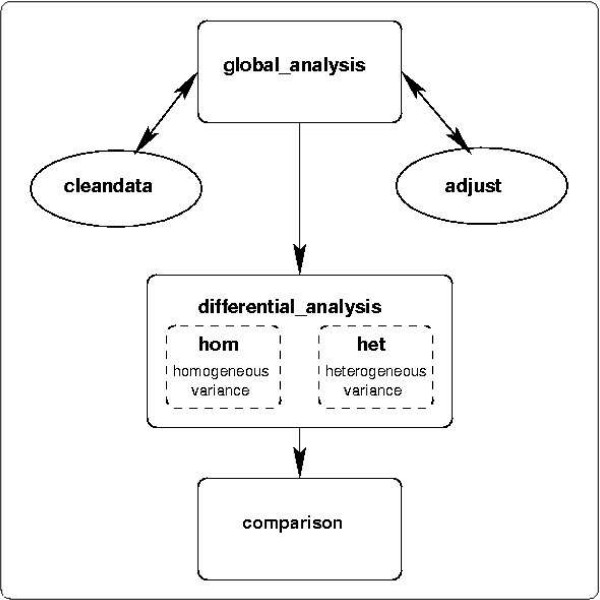
**AnovArray Package. **AnovArray package: map of the use of the major macros.

The five macros of the package can be used either independently or in a concerted way as indicated in the strategy analysis described in figure 1. The anova model is defined in the macro **global_analysis **by the user. This macro computes the classical anova table which permits to identify factors which are important to explain observed differences in gene expression. As explained in the previous section, several graphs described in figure 2 are available to check model assumptions: variance homogeneity and gaussian distribution of residuals. These graphs can also be very useful to highlight which experimental factor affects a sub-population of genes. Several models can be tested and the quality control facilities (statistics in the table of anova, graphs) permit to select which one is the more accurate. Depending on the results given by the macro global_analysis, it could be necessary to use macros **adjust **and **cleandata**. The macro adjust will then permit to systematically remove undesirable effects (factors) observed in graphs obtained by the macro global_analysis. In the same manner, the macro cleandata makes it possible to remove genes which do not respect the assumptions of the model. We advise to use this iterative process (global_analysis, cleandata and adjust) before using the macro **differential_analysis**. The aim of this process is to make sure that data are well fitted by the model and that model assumptions are satisfied. This process is very important to get reliable results on differentially expressed genes.

As explained in the previous section, the package also permits the differential analysis under two hypotheses: either genes have equal variance (homogeneous model – HOM) or each gene has its own variance (heterogeneous model – HET). The macro differential_analysis produces the list of genes differentially expressed between several experimental conditions using p-values and adjusted p-values statistics. A p-value is defined as the probability of rejecting the null hypothesis {The interaction gene x condition is null.}, if true. P-values are calculated for each gene under the hypothesis that all genes have the same variance and under the hypothesis that each gene has its own variance. By using the correction for multiple comparisons FDR [7](False Discovery Rate), a gene is differentially expressed if its adjusted p-value is lower than a significance level given by the user.

Finally, the macro **comparison **enables to compare graphically the results obtained by the two models of variance. In a way, the plot of adjusted p-values under hypothesis of homogeneous variance versus adjusted p-values under hypothesis of heterogeneous variance indicates the genes which probably do not satisfy the homogeneity of variance hypothesis.

To summarize AnovArray package permits successive usage of different anova models (principal effects and their interactions) in order to construct an adaptive approach of gene expression data analysis.

## Results and discussion

AnovArray has been used for the analysis of a macroarray dataset resulting on the hybridisation of 72 membranes. This dataset contains the level of expression of 1920 bovine embryonic cDNA pieces under three conditions of complex sample preparation into two tissues (ovary, brain). The package turned out to be useful and rapid to identify differentially expressed genes between both tissues and three protocols of complex sample preparation (Degrelle et al., manuscrit in preparation). In particular, the anova model emphasizes the existence of an interaction between gene and sample preparation method, between gene and tissue and between gene and sample and tissue. This analysis highlights that the sample preparation could affect differential analysis results.

The dataset described in the manual is a subset of the previous one. The frame of the experiment is conserved and only hybridisation on six membranes have been retained. Three samples obtained from bovine tissues A and B are hybridised on one macroarray membrane. This dataset containing 1525 cDNA was created to give an usage of the AnovArray package. The aim of this analysis is the detection of genes differentially expressed between tissues. The anova model used is

**Y**_*tgi *_= *μ *+ *α*_*t *_+ *β*_*g *_+ *γ*_*tg *_+ *ε*_*tgi*_

where *Y*_*tgi *_is the expression (transformed by logarithm in base 2) of the *i*^*th *^observation of the gene g in tissue t, *μ *is the mean effect, *α*_*t *_is the effect of tissue t with t in {A, B}, *β*_*g *_is the effect of gene g with 1525 levels, *γ*_*tg *_the interaction between tissue t and gene g, and *ε*_*tgi *_is the residual error. The model assumes that the residuals *ε*_*tgi *_are independent and normally distributed with equal variance and mean zero (*ε*_*tgi *_~  (0, *σ*^2^)) if variance is homogeneous, or (*ε*_*tgi *_~  (0, )) if variance is heterogeneous. To perform a differential analysis, we test the null hypothesis {the interaction *γ*_*tg *_is null}. The Fisher statistic is calculated in homogeneous (resp. heterogeneous) model using the variance *σ*^2 ^(resp. ). The power of the Fisher test depends on the accuracy of the variance estimation and at least six measures are necessary to estimate . Two genes are found differentially expressed between the two tissues with the hypothesis of homogeneity of variance and none with the hypothesis of heterogeneity of variance. Methods and statistics are described in a user's guide available at .

## Conclusion

We have presented a tool for analysis microaray and macroarray based on the analysis of variance. This package contains some useful graphs to describe and analyse microarray and macroarray data. It permits to evaluate the source of bias, the assumptions underlying the model (distribution of residuals, distribution of variances). It gives also the list of differentially expressed genes between more than two conditions using the false discovery rate (FDR).

Our future development will concern an extension to mixed models and an addition of other multiple correction methods.

## Availability and requirements

**Project name: **AnovArray: a set of SAS macros for the analysis of variance of gene expression data.

**Project home page: **

**Operating system(s): **Platform independent

**Programming language: **SAS^®^

**Other Requirements: **SAS^® ^release 8.01 with modules BASE SAS, SAS/Stat and SAS/Graph.

Licence:

**Any restrictions to use by non-academic user: **citation

## Authors' contributions

KP developed the software. CHA, HC, FR and SR conceived the study, participated in its design and coordination. SD, IH, JPR provided experiment dataset and tested software.
